# Methylene blue for malaria in Africa: results from a dose-finding study in combination with chloroquine

**DOI:** 10.1186/1475-2875-5-84

**Published:** 2006-10-08

**Authors:** Peter E Meissner, Germain Mandi, Boubacar Coulibaly, Steffen Witte, Théophile Tapsoba, Ulrich Mansmann, Jens Rengelshausen, Wolfgang Schiek, Albrecht Jahn, Ingeborg Walter-Sack, Gerd Mikus, Jürgen Burhenne, Klaus-Dieter Riedel, R Heiner Schirmer, Bocar Kouyaté, Olaf Müller

**Affiliations:** 1 Department of Tropical Hygiene and Public Health, Ruprecht-Karls-University, INF 324, 69120 Heidelberg, Germany; 2 Department of Paediatrics IV Neonatology, Ruprecht-Karls-University, INF 150, 69120 Heidelberg, Germany; 3 Centre de Recherche en Santé de Nouna, PO Box 02, Nouna, Burkina Faso; 4 Institute of Medical Biometrics and Informatics, Ruprecht-Karls-University, Heidelberg, Germany; 5 Institute of Medical Informatics, Biostatistics, and Epidemiology, University of Munich, Munich, Germany; 6 Department of Internal Medicine VI, Clinical Pharmacology and Pharmacoepidemiology, Ruprecht-Karls-University, Heidelberg, Germany; 7 DSM Fine Chemicals, Linz, Austria; 8 Biochemistry Center, Ruprecht-Karls-University, Heidelberg, Germany

## Abstract

The development of safe, effective and affordable drug combinations against malaria in Africa is a public health priority. Methylene blue (MB) has a similar mode of action as chloroquine (CQ) and has moreover been shown to selectively inhibit the *Plasmodium falciparum *glutathione reductase. In 2004, an uncontrolled dose-finding study on the combination MB-CQ was performed in 435 young children with uncomplicated falciparum malaria in Burkina Faso (CQ monotherapy had a > 50% clinical failure rate in this area in 2003). Three serious adverse events (SAE) occurred of which one was probably attributable to the study medication. In the per protocol safety analysis, there were no dose specific effects. The overall clinical and parasitological failure rates by day 14 were 10% [95% CI (7.5%, 14.0%)] and 24% [95% CI (19.4%, 28.3%)], respectively. MB appears to have efficacy against malaria, but the combination of CQ-MB is clearly not effective in the treatment of malaria in Africa.

## Background

The increasing resistance of *Plasmodium falciparum *to existing safe and affordable drugs such as chloroquine (CQ) and pyrimethamine-sulfadoxine severely threatens the available options for malaria control in sub-Saharan Africa (SSA) [1]. To maximise efficacy and to minimise resistance development, malaria combination therapy has become a new paradigm [2]. Although a small number of new malaria drugs including artemisinin derivates have been developed in recent years, these are usually too expensive for unsubsidised use in SSA.

Methylene blue (MB) has already been used some 100 years ago against malaria but it disappeared when CQ and other drugs entered the market [3]. MB, a specific inhibitor of *P. falciparum *glutathione reductase, has the potential to reverse CQ resistance and it prevents the polymerization of haem into haemozoin similar to 4-amino-quinoline antimalarials [4]. It has recently been shown that the combination MB-CQ is safe in adults and children with and without G6PD deficiency [5-7]. However, oral MB given twice daily (4 mg/kg/day) together with a standard dose of CQ over three days was not effective in the treatment of uncomplicated malaria in young children of Nouna town in Burkina Faso in 2003. The day 14 CF rate was 53.7%, 95% CI 37.4–69.3, in the CQ control arm [7]. The aim of the present study was to assess the safety and efficacy of higher and more frequent MB doses in combination with CQ in a comparable study population in the same area.

## Materials and methods

A single centre uncontrolled trial with three dose levels was conducted during the rainy season 2004 at the district hospital of Nouna in north-western Burkina Faso, an area of intense malaria transmission [8]. Febrile children from Nouna town were invited to the hospital for examination and treatment. Inclusion criteria were age 6–59 months, uncomplicated malaria (axillary temperature ≥ 37.5°C and ≥ 2.000 *P. falciparum *asexual parasites per μl blood), haemoglobin ≥ 8 g/dl, absence of severe malaria and other significant disease, and informed written consent. The study was conducted in accordance with the internationally established principles for GCP and controlled by a data safety monitoring board (DSMB). The protocol was approved by the Ethics Committee of the University of Heidelberg and the local Ethics Committee in Burkina Faso.

Children were recruited for the three dose levels sequentially. In addition to receiving a total CQ dose of 25 mg/kg (10 mg/kg on days 0 and 1, and 5 mg/kg on day 2), study children received total doses of MB of 36, 54, and 72 mg/kg respectively. At each dose level children were block-randomized by envelope to two or four MB doses per day. MB (Mayrhofer Pharmazeutika, Linz/Austria) was given as a 2.3% solution with fruit flavouring and honey supplement to mask the bitter taste. CQ (tablets or syrup) was taken from the essential drug stock of the hospital. In case of vomiting within 30 minutes after intake, the drugs were re-administered once.

The dose escalation process for a dosage regimen went into the next higher dosage level if the safety (i.e. one-sided 95% CI for the incidence of relevant adverse events below 0.1) and the efficacy criterion (i.e. one-sided 95% CI for the incidence of CF below 0.15) were fulfilled at most in one dosage level.

Study participants were hospitalized for 72 hours. Treatment failures were managed according to national guidelines. Children were systematically examined on day 0, 1, 2, 3, 4 and 14. Blood samples were processed with standard methods in the laboratory of the *Centre de Recherche en Santé de Nouna *(CRSN) [6]. Methaemoglobin formation was monitored twice daily on day 0 and once on day 1, 2 and 3. Other laboratory parameters like liver enzymes, serum creatinine or the phenotypical G6PD status were available at any time if clinically indicated. Based on filter paper blood samples, the G6PD genotype was determined in Germany [9].

Treatment outcomes were classified according to the WHO guidelines from 2003 as adequate clinical and parasitological response (ACPR), early treatment failure (ETF), late clinical failure (LCF), late parasitological failure (LPF) and clinical failure (CF = ETF+LCF) [10].

Assuming a 10% drop-out rate, 72 patients per group and dose level were needed to discover relevant safety and efficacy scenarios with a power of 80% and to avoid a false positive dose effect with probability of 95%. The sample size estimation assumed the independence of the efficacy outcome and the safety outcome. The null hypothesis had to be rejected in each level if the incidence of the relevant study adverse events fell below 10%. For treatment outcomes, the null hypothesis was rejected if the CF rate was below 15%. The safety analysis was based on the children who have received at least one dose of CQ-MB (FAS = full analysis set). Efficacy data were assessed in the population of children who received the full course of treatment (per protocol population, PP). All data were double entered and SAS^® ^8.2 was used to analyse the data. Continuous variables in two groups were compared with the nonparametric Wilcoxon-Mann-Whitney (WMW) or Kruskal-Wallis Test (KW), categorical variables with Chi-square-test (Chi). The clinical failure rates were analysed using the two fixed factors (group and level) in a logistic regression model, likelihood ratio tests (LR) were conducted.

## Results

**Figure 1:**
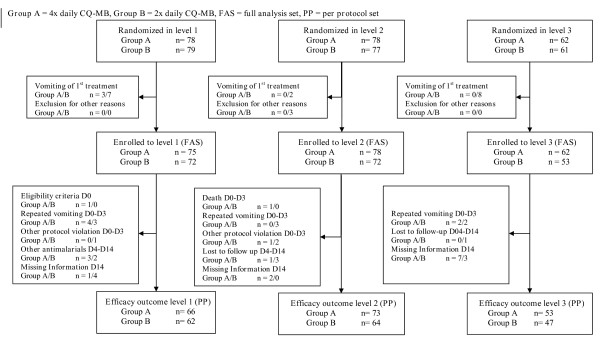
Trial profile.

Overall, 435 children were included into the study, 412 in the FAS analysis and 364 in the PP analysis (Figure 1). The lower number in level 3 is explained by an insufficient number of malaria cases occurring at the end of the rainy season. There were no significant differences in baseline parameters between the two randomized groups. Between the three dose levels there were differences in prior treatment (37.4%, 30.0%, 21.7%, p_Chi _= 0.0233), age (31.6, 32.1, 36.9 months, p_KW _= 0.0124), and in methaemoglobin (1.5, 1.1, 1.1%, p_KW _< 0.0001). Overall 88/409 (21.5%) of FAS children were found to be genotypically G6PD deficient (50 heterozygote, 38 homo- or hemizygote).

There were three serious adverse events in 412 patients (0.7%, 95% CI 0.2–2.1). During dose level 2, one child progressed to severe malaria within 24 hours of inclusion into the study and one child died of diarrhoea on day 8. During dose level 3, in one G6PD deficient male child the Hb dropped from 8.7 g/dl at inclusion to 4.7 g/dl on day5 but improved afterwards on iron supplementation. Clinically there were no signs of haemolysis and total serum bilirubin was normal on day 3. In seven other children the Hb value dropped by more than 3 g/dl, three of these were found to be G6PD deficient. There were no major differences in the incidence of other adverse events between study groups and dose levels (data not shown).

Efficacy outcomes are given in Table 1. The overall day 14 CF and LPF rates were 38/364 (10.4%, 95% CI 7.5–14.0) and 86/364 (23.6%, 95% CI 19.4–28.3) respectively without interactions between groups and drug levels (see Table 1).

**Table 1:** Efficacy results by group and dose level (per protocol analysis)

	2× daily	4× daily	Group comparison
Level1 MB 36 mg/kg			
- ETF/N	1.6%, 1/62	3.0%, 2/66	p = 0.5923 (1)
- LCF14/N	8.1%, 5/62	3.0%, 2/66	p = 0.2047 (1)
- TF14/N	9.7%, 6/62	6.1%, 4/66	p = 0.4452 (1)
- LPF14/N	24.2%, 15/62	22.7%, 15/66	p = 0.8449 (1)
Level2 MB 54 mg/kg			
- ETF/N	3.1%, 2/64	6.9%, 5/73	p = 0.3140 (1)
- LCF14/N	4.7%, 3/64	11.0%, 8/73	p = 0.1688 (1)
- TF14/N	7.8%, 5/64	17.8%, 13/73	p = 0.0783 (1)
- LPF14/N	35.9%, 23/64	17.8%, 13/73	p = 0.0159 (1)
Level3 MB 72 mg/kg			
- ETF/N	0.0%, 0/47	0.0%, 0/52	-
- LCF14/N	10.6%, 5/47	9.6%, 5/52	p = 0.8661 (1)
- TF14/N	10.6%, 5/47	9.6%, 5/52	p = 0.8661 (1)
- LPF14/N	21.3%, 10/47	19.2%, 10/52	p = 0.8002 (1)
Level comparison			
- p-value (ETF)	0.3266 (2)	0.0595 (2)	0.7290 (3), 0.0960 (4), 0.9930 (5)
- p-value (LCF14)	0.4824 (2)	0.1492 (2)	0.3168 (3), 0.2613 (4), 0.1722 (5)
- p-value (LF14)	0.8684 (2)	0.0838 (2)	0.2944 (3), 0.2634 (4), 0.1984 (5)
- p-value (LPF14)	0.1764 (2)	0.7631 (2)	0.1155 (3), 0.4045 (4), 0.2486 (5)

ETF = early treatment failure, LCF14 = late clinical failure (day 14), TF14 = treatment failure (ETF or LCF14), LPF14 = late parasitological failure (day 14), (1) group comparisons within level, (2) level comparison within group, (3) test of overall group comparison, (4) test of overall level comparison, (5) test of group*level interaction, Likelihood ratio tests were used

## Discussion

The results of this study provide some indirect evidence that MB, a cheap drug which is registered in most countries, could be effective as treatment of uncomplicated malaria in SSA. Compared to the low efficacy demonstrated in a previous study where a low dosage of MB (12 mg per kg over three days) in combination with CQ was used, the CQ-MB combination in this study appeared to be more effective at three to six times higher MB doses (36–72 mg per kg over three days) [7]. However, although the present study was done during a comparable time period and in a comparable study population, the results of these two studies are not fully comparable due to possibly different incidences of other febrile diseases as well as differences in malaria transmission intensity between the year 2003 and 2004.

There were no differences in efficacy between a two times and a four times daily regimen in this dose-finding study; this suggests that a more than twice daily MB regimen would have no benefits. MB has a rather short half-life which has been estimated at 5–6 hours [4]. However, our own data point to a slightly longer half-life of 15 hours (Walter-Sack, unpublished). As artemisinin drugs have an even shorter half-life and have been shown to be effective with once daily regimens, a once daily regimen of MB may also be sufficient [1,11,2]. This could be clarified in future studies.

Methylene blue belongs to a group of drugs considered to potentially cause haemolysis when given to persons with G6PD deficiency [12]. However, in most of SSA, the class III G6PD deficiency dominates, where there remains an enzyme activity of 15–25%, compared to only 0–5% in class II deficiency [12]. It is reassuring that there was no evidence for haemolysis being a major side effect in an overall group of 593 children treated with MB during the 2003 and 2004 studies in Burkina Faso, which included a total of 112 children with G6PD deficiency [7]. However, in one child of this dose finding study an episode of haemolysis may have occurred and could have been attributed to MB. Only larger studies would be able to fully quantify this hypothetical risk of treating malaria in African children with MB. This also concerns other drugs which may lead to haemolysis in G6PD deficiency, such as dapsone, a drug recently approved for malaria therapy in combination with chloroproguanil [13].

Given the potential of CQ resistance reversal, the combination of CQ with MB was studied in trials conducted in Burkina Faso [4,6,7]. However, this study has clearly shown that the CQ-MB combination was not sufficiently effective even at higher MB doses. As follow-up was only for 14 days, it can be expected that a 28 day follow-up would have shown even higher clinical and parasitological failure rates. A factor that has probably contributed to this result is the (previously unknown) high CQ background resistance in the urban Nouna study area [7]. In analogy to the artemisinin combination schemes, MB may need to be combined with a locally effective partner drug when given in a three day regimen [1,2,11]. Moreover, it has recently been shown that CQ is antagonistic to MB when combined against *P. falciparum *in vitro, which may further explain the impairment of efficacy observed [14].

In conclusion, this study has provided indirect evidence for efficacy of MB in the treatment of uncomplicated falciparum malaria in SSA but has clearly shown that the combination CQ-MB is not useful in the treatment of malaria in SSA.

## Authors' contributions

P Meissner and G Mandi contributed equally to the study. P Meissner, G Mandi, S Witte, U Mansmann, A Jahn, I Walter-Sack, H Schirmer and O Müller designed the study. P Meissner, G Mandi, B Coulibaly, J Rengelshausen, W Schiek, G Mikus, J Burhenne and KD Riedel conducted the laboratory and clinical work. S Witte, U Mansmann and T Tapsoba did the statistical analysis. All authors contributed to the writing of the paper. O Müller was the principal investigator.

## Conflict of interest statement

All authors have no commercial or other association that might pose a conflict of interest. W. Schiek is employed by DSM. The sponsors of the study had no role in study design, data collection and data analysis. The authors had full access to all the data and the corresponding author had final responsibility for the decision to submit for publication.
